# HPV16 E6 enhances the radiosensitivity in HPV-positive human head and neck squamous cell carcinoma by regulating the miR-27a-3p/SMG1 axis

**DOI:** 10.1186/s13027-021-00397-w

**Published:** 2021-08-13

**Authors:** Dan Long, Li Xu, Zeyi Deng, Dandan Guo, Yangchun Zhang, Zhaohui Liu, Chunlin Zhang

**Affiliations:** 1 Department of Otorhinolaryngology, Head and Neck Surgery, Affiliated Hospital of Zunyi Medical University, Zunyi, 563000 Guizhou China; 2 Department of Otorhinolaryngology, Head and Neck Surgery, Zhujiang Hospital, Southern Medical University, Guangzhou, 510000 China

**Keywords:** Head and neck cancer, HPV16, Radiosensitivity, E6, miR-27a-3p, SMG1

## Abstract

**Background:**

Head and neck squamous cell carcinoma (HNSCC) is the 6th most common malignant cancer type worldwide. Radiosensitivity has been shown to be significantly increased in patients with human papillomavirus (HPV)-positive HNSCC compared with HPV-negative patients. However, the clinical significance of HPV and its regulatory mechanisms in HNSCC are largely unknown. The aim of our study was to explore the regulatory mechanism of miR-27a-3p in the radiosensitivity of HPV-positive HNSCC cells.

**Methods:**

E6-overexpressing and E6-knockdown HNSCC cell lines were generated and the transfection efficiencies were evaluated by quantitative real-time PCR (RT-qPCR) and western blotting. The expression of miR-27a-3p and DiGeorge syndrome critical region 8 (DGCR8) was examined by RT-qPCR after transfection with E6 overexpressing plasmid or E6 siRNA. The effects of miR-27a-3p on the radiosensitivity of HNSCC cells were explored by a colony formation and TUNEL staining assays. Bioinformatic tools and luciferase reporter assays were used to identify that SMG1 is the direct target of miR-27a-3p. Furthermore, the effect of E6 overexpression on the regulation of the miR-27a-3p/SMG1 axis was investigated.

**Results:**

In our study, we found overexpression of HPV E6 upregulated the expression of DGCR8 and miR-27a-3p in HNSCC cells. We next confirmed that DGCR8 positively regulated the expression of miR-27a-3p in HNSCC cells. The luciferase reporter gene results verified that miR-27a-3p targeted the 3’UTR of SMG1 mRNA. MiR-27a-3p mimics transfection resulted in a decrease in SMG1 expression and miR-27a-3p inhibitor transfection increased SMG1 expression. Apoptotic activity of HNSCC cells was significantly increased in miR-27a-3p mimics HNSCC cells compared with control HNSCC cells. After treatment with 4 Gy irradiation, UM-SCC47 cells transfected with miR-27a-3p inhibitor or SMG1 overexpressing plasmid formed more colonies than the corresponding control cells. Furthermore, the rescue experiments demonstrated that HPV16 E6 improved the radiosensitivity of HNSCC cells by targeting miR-27a-3p/SMG1.

**Conclusion:**

Our study demonstrated that HPV16 E6 activated the DGCR8/miR-27a-3p/SMG1 axis to enhance the radiosensitivity. Our findings might provide a novel therapeutic target to improve the response of HNSCC to radiotherapy.

## Introduction

Cancer ranks as the leading or second leading cause of premature death in almost 100 countries worldwide, and in men, lip and oral cavity cancer ranks third in transitioning countries [1]. Head and neck squamous cell carcinoma (HNSCC) can be divided into tongue squamous cell carcinoma (TSCC), oral squamous cell carcinoma (OSCC), laryngeal squamous cell carcinoma (LSCC) and nasopharyngeal carcinoma (NPC) according to the tumor site [2]. Alcohol abuse, and smoking are generally considered to be the historical risk factors for HNSCC, however, high-risk HPV is also a pathogenic factor for the initiation and progression of HNSCC [3]. HPV-positive HNSCC cases have increased in recent years [4], and it is estimated that 31% of patients with oropharyngeal cancer, a subtype of HNSCC, test positive for HPV infection [5, 6]. Patients with HPV-positive HNSCC have a better outcome in terms of both overall survival and reduced risk of recurrence than those with HPV-negative HNSCC [7].

HPV is a small, nonenveloped double-stranded DNA virus [8]. Among the high-risk HPV types, HPV16 and HPV18 are major viral carcinogens, accounting for the development of up to nearly 5% of the total cancer incidence worldwide [9, 10]. HPV E6 and E7 are two major oncoproteins encoded by HPV 16 and 18 [11], which have been shown to promote tumorigenesis by inducing cell immortality and migration, changing the cell cycle, controlling apoptosis, and fostering avoidance of host immune surveillance [12, 13]. The E6 protein of high-risk HPV strains induces ubiquitination-mediated degradation of p53, disrupting its tumor suppressor activity [14]. Moreover, E6 promotes cell proliferation, apoptosis resistance and invasion by activating the NF-κB and Akt pathways in HNSCC [15]. HPV E6 can also upregulate the expression of heparanase which was shown to be responsible for the invasive phenotype of HNSCC [16]. HPV-positive HNSCC has been identified as a subset of HNSCC distinct from HPV- negative HNSCC in terms of tumor biology and clinical characteristics, including its improved radiosensitivity [17]. In our previous study, we found that HPV16 E6 was associated with the radiosensitivity in HPV-positive HNSCC [18]. However, the mechanism by which the HPV E6 protein regulates radiosensitivity in HNSCC is still not fully understood.

MiRNAs play an important role in the occurrence and development of human cancers [19]. MiR-27a-3p was shown to function as an oncogene by enhancing the expression of cyclin D1, which led to the promotion of cell cycle progression and proliferation in bladder cancer cells [20]. It has been reported that miR-27a-3p regulated the proliferation and apoptosis of cervical cancer, gastric cancer and colon cancer cells [21, 22]. Furthermore, miR-27a-3p plays a major role in tumorigenesis, and upregulates observed in OSCC [23, 24]. SMG1 is a member of the phosphoinositol kinase-like kinase family and has been shown to function as a tumor suppressor gene [25]. It was reported that SMG1 was involved in the EMT process and tumorigenesis in multiple cancers [26]. In our previous study, we found that SMG1 increased the sensitivity of HNSCC cells to radiotherapy and that E6 downregulated SMG1 expression via DNMT1 [18]. However, the connection between miR-27a-3p/SMG1 and their roles in HNSCC remains unclear. In our study, we proved that HPV16 E6 promoted the expression of DGCR8, which in turn increased the expression of miR-27a-3p. Gene SMG1 was identified as the downstream target of miR-27a-3p. Collectively, our findings showed that HPV16 E6 improved radiosensitivity in HPV-positive HNSCC by regulating the miR-27a/SMG1 axis.

## Materials and methods

### Cell cultures

The HPV16-negative HNSCC cell lines FaDu and UM-SCC-4 and the HPV16-positive HNSCC cell lines UPCI-SCC-090 and UM-SCC47, which were purchased from ATCC, were used in this study. FaDu and UM-SCC-47 cells were cultured in DMEM (Gibco, Carlsbad, CA) supplemented with 10% fetal bovine serum (FBS). UM-SCC-4 cells were cultured in DMEM/F12 medium (Gibco, Carlsbad, CA) supplemented with 10% FBS. UPCI-SCC-090 cells were cultured in MEM (Gibco, Carlsbad, CA) supplemented with 10% FBS. All cells were cultured in an incubator at 37 °C in 5% CO_2_. Cells were acclimated for 24 h before any treatments in all experiments.

### Cell transfection

SiRNAs targeting E6 and DGCR8 and the corresponding control siRNAs, as well as the miR-27a-3p mimics, miR-27a-3p inhibitors and corresponding miR-controls were purchased from RiboBio (Guangzhou, China). Cells were transiently transfected with 50 nM siRNA using Lipofectamine 3000 (Invitrogen, USA). MiR-27a-3p mimics, miR-27a-3p inhibitors and miR-controls were transfected into cells at a concentration of 40 nM using Lipofectamine 3000. The plasmids pcDNA-E6, pcDNA-DGCR8, and pcDNA-SMG1 were obtained from iGenBio (Guangzhou, China) and transfected into HNSCC cell lines using Lipofectamine 3000 following the manufacturer’s protocol. The siRNA sequences are listed in Table 1.

**Table 1:** Sequences of siRNAs against specific targets

Gene	Sequences
si-DGCR8	guide strand: UCAUUUUCUUAUAAUGCAGAC passenger strand: CUGCAUUAUAAGAAAAUGAAG
si-E6	guide strand: AAUCGUAAACACACUUUACAU passenger strand: GUAAAGUGUGUUUACGAUUGC

### RNA extraction and real-time quantitative PCR analysis

Total RNA was extracted using TRIzol reagent (Invitrogen, Carlsbad, CA), and first-strand cDNA was generated using PrimeScript RT Master Mix (Takara, Japan) and either gene-specific primers or random primers. Then, SYBR Green-based RT-qPCR was performed using gene-specific primers. GAPDH was used as the loading control for normalization of mRNA expression levels, and U6 was used as the loading control for normalization of miRNA expression levels. The 2-ΔΔCt method was used for relative quantitation of gene expression levels. The primers sequences are listed in Table 2.

**Table 2:** The primer sequences used for RT-qPCR

Gene	Primer
HPV16 E6	Forward: CTGCAATGTTTCAGGACCCAC Reverse: GTTGTTTGCAGCTCTGTGCAT
DGCR8	Forward: GTGCATGCTTGTCCCTTTGG Reverse: TGCCAACATACCTCGTAGGG
miR-27a-3p	Forward: CAGTTCACAGTGGCTAAGTTC Reverse: CAGTTTTTTTTTTTTTTTGCGGAA
SMG1	Forward: AGTTAATGGAGGCCACACCC Reverse: ACTCTAAGGCTTTTACCTTTTTCAA
GAPDH	Forward: AATCCCATCACCATCTTCC Reverse: ATCCGTTGACTCCGACCTTCAC
U6	Forward: GAGTCCTTCCACGATACCAA Reverse: AACGCT TCACGAATT TGC

### Protein extraction and western blot analysis

Homogenized adherent cells were lysed using RIPA buffer (KeyGene Biotech, China). Proteins (30 μg) were separated using SDS-PAGE and were then transferred to polyvinylidene difluoride membranes. The membranes were washed, blocked, and incubated with primary antibodies specific for E6 (dilution 1:1000; ab70, Abcam, USA), DGCR8 (dilution 1:1000; 10996-1-AP, Proteintech, China), SMG1 (dilution 1:1000; Q25, CST, UK) and GAPDH (dilution 1:5000; 1E6D9, Proteintech, China) prior to incubation with horseradish peroxidase-conjugated secondary antibodies (dilution 1:10000; BA1051/BA1055, BOSTER, China). Immunocomplexes were visualized using an enhanced chemiluminescence assay kit (BeyoECL Plus, Beyotime, China).

### Immunofluorescence assay

For immunofluorescence analysis, HNSCC cells were plated on coverslips in a 24-well cell culture plate (Corning, NY, USA). After transfection with E6 overexpressing or interference plasmids for 48 h, the cells were fixed with 4% paraformaldehyde (Beyotime, Jiangsu, China) at room temperature for 10 min, permeabilized with 0.5% Triton X-100 for 20 min, washed twice in PBS, and blocked with 3% BSA (KeyGene Biotech, China) in PBS. After 1 h, cells were incubated with the anti-DGCR8 antibody (dilution 1:1000, 10996-1-AP, Proteintech, China) at 4 °C overnight and then with a FITC-conjugated secondary antibody (dilution 1:200, SA00013-2, Proteintech, China) for 1 h and with DAPI (KeyGene Biotech, China) to stain nuclei. Fluorescence was observed under a fluorescence microscope (Olympus IX81, Japan).

### TUNEL assay

A TUNEL kit (Beyotime, Jiangsu, China) was used for the terminal deoxynucleotide transferase-mediated nickel labeling (TUNEL) assay, and apoptosis levels were evaluated according to the manufacturer’s instructions. After TUNEL, nuclei were labeled with 4′,6-diamidino-2-phenylindole (DAPI, Invitrogen, California, USA), and TUNEL-positive cells were observed under a fluorescence microscope (Olympus IX81, Japan). The average number of apoptotic cells in each group was calculated by averaging the number of TUNEL-positive apoptotic cells in 10 fields at 200× magnification.

### Colony formation assay

Cells were seeded into a 6-well plate at a density of 0.5–1 × 10^3^ and were then cultured for 14 days in 5% CO_2_ at a temperature of 37 °C. After the incubation period, the culture medium was discarded, and the cells were fixed with 4% polyoxymethylene (Beyotime, Jiangsu, China), stained with 0.1% crystal violet staining solution (Beyotime, Jiangsu, China) for 20 min. The colony clusters were counted by macroscopic observation, and the number of colonies was used to evaluate the colony formation ability.

### Dual luciferase reporter assay

Using the TargetScan database (http://www.targetscan.org/vert_72/), we found potential binding sites between SMG1 and miR-27a. The wild-type or mutant SMG1 fragment containing the predicted binding sites of miR-27a was subcloned into the psiCHECK2 dual-luciferase vector (Promega, USA). The luciferase reporter plasmids were cotransfected into HNSCC cells with the miR-27a mimic or the negative control. The relative luciferase activity was measured with a Dual-Luciferase Reporter Assay System (Promega, USA) according to the manufacturer’s instructions.

### Statistical analysis

SPSS 21.0 (IBM, USA) was adopted for statistical analysis. Data was shown as the mean ± standard deviation (mean ± SD) values. Two-tailed Student’s t test was used to analyze differences between the groups and a *P* value < 0.05 was considered statistically significant. All experiments were repeated in triplicate.

## Results

### HPV16 E6 upregulated the expression of DGCR8 in HNSCC cells

**Fig. 1:**
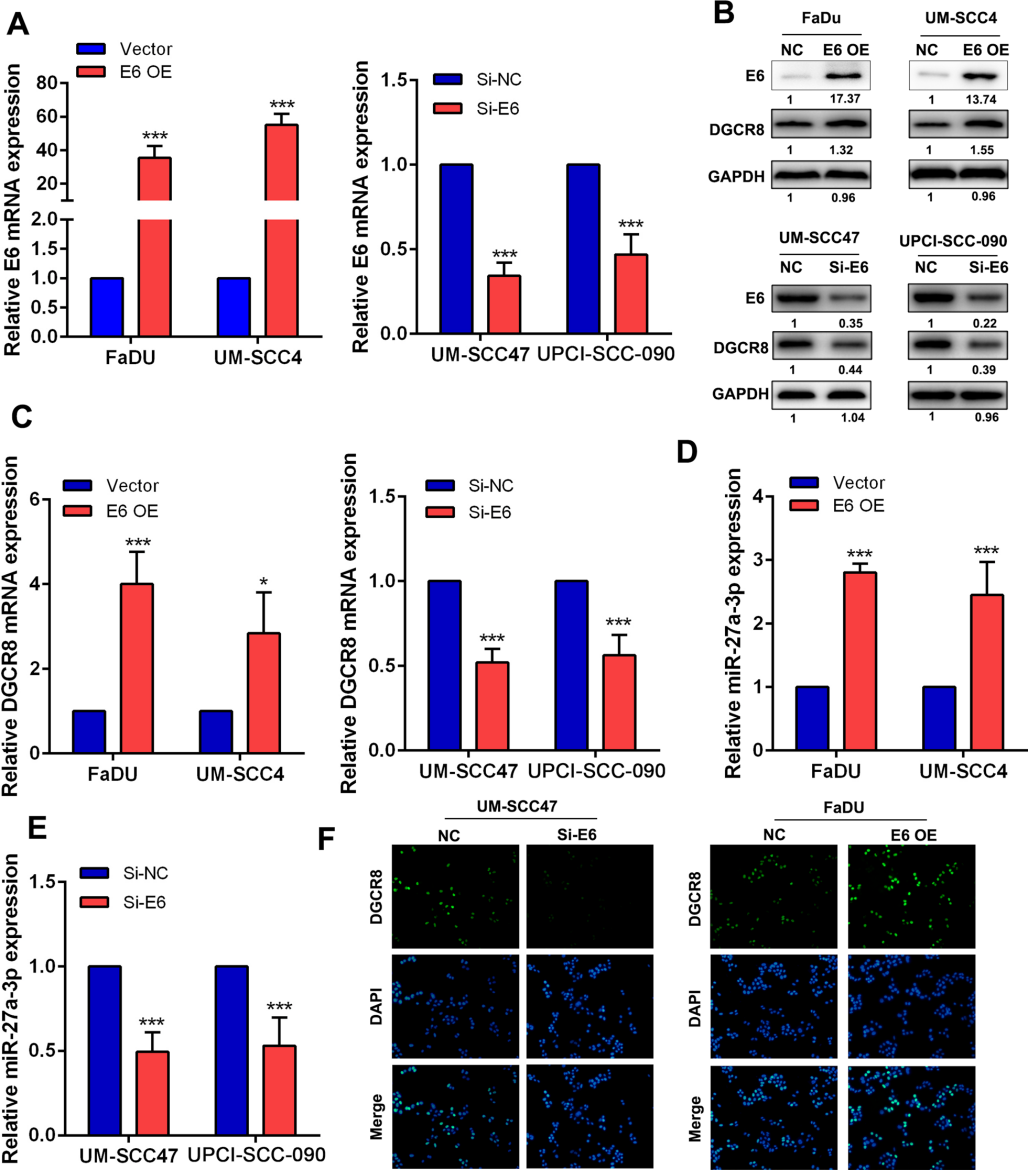
HPV16 E6 regulated the expression of DGCR8 in HNSCC cells. **a** The relative mRNA expression level of HPV16 E6 in E6-overexpressing/knockdown HNSCC cell lines. **b** The relative protein expression level of HPV E6 and DGCR8 in E6-overexpressing/knockdown HNSCC cell lines. **c** The relative mRNA expression level of DGCR8 in E6-overexpressing/knockdown HNSCC cell lines. **d**, **e** The relative expression level of miR-27a-3p in E6-overexpressing/knockdown HNSCC cell lines. **f** The expression sites of DGCR8 in E6-overexpressing HNSCC cell lines were observed by immunofluorescence. Original magnification ×200. Data were presented as mean ± SD. *P < 0.05; **P < 0.01; ***P < 0.001

In our previous study, we found that HPV16 E6 was significantly higher in HPV-positive HNSCC cells than in HPV-negative cells [18]. We also found that DGCR8, an RNA binding protein facilitating the maturation of miRNAs, was expressed at higher levels in UM-SCC47/UPCI-SCC-090 cells than in FaDu/UM-SCC4 cells [27]. To further explore the regulatory role of HPV E6 in HNSCC based on the expression level of HPV E6 in 4 HNSCC cell lines, we generated an E6-overexpressing cell line with FaDu/UM-SCC4 cells and an E6-knockdown cell line with UM-SCC47/UPCI-SCC-090 cells in this study. The overexpression and knockdown efficiencies were evaluated at the mRNA and protein levels using RT-qPCR and western blotting, respectively. The expression of E6 was significantly upregulated in E6-overexpressing cells and downregulated in E6-knockdown cells (Fig. 1a). Interestingly, overexpression of HPV E6 upregulated the expression of DGCR8, while HPV E6 knockdown decreased the expression level of DGCR8, suggesting that HPV E6 played a regulatory role in DGCR8 expression (Fig. 1b, c). MiR-27a-3p has been reported to be associated with tumor radiosensitive. Surprisingly, we found that the expression of miR-27a-3p was upregulated in E6-overexpressing HNSCC cell lines and downregulated in E6-knockdown HNSCC cell lines (Fig. 1d, e). By the immunofluorescence assay, we also confirmed that upregulation of HPV E6 increased the level of DGCR8 (Fig. 1f). Collectively, these findings indicated that HPV E6 induced the expression of DGCR8 and miR-27a-3 in HNSCC.

### *HPV16 E6 upregulated miR-27a-3p *via* DGCR8 and miR-27a-3p improved radiosensitivity in HNSCC*

**Fig. 2:**
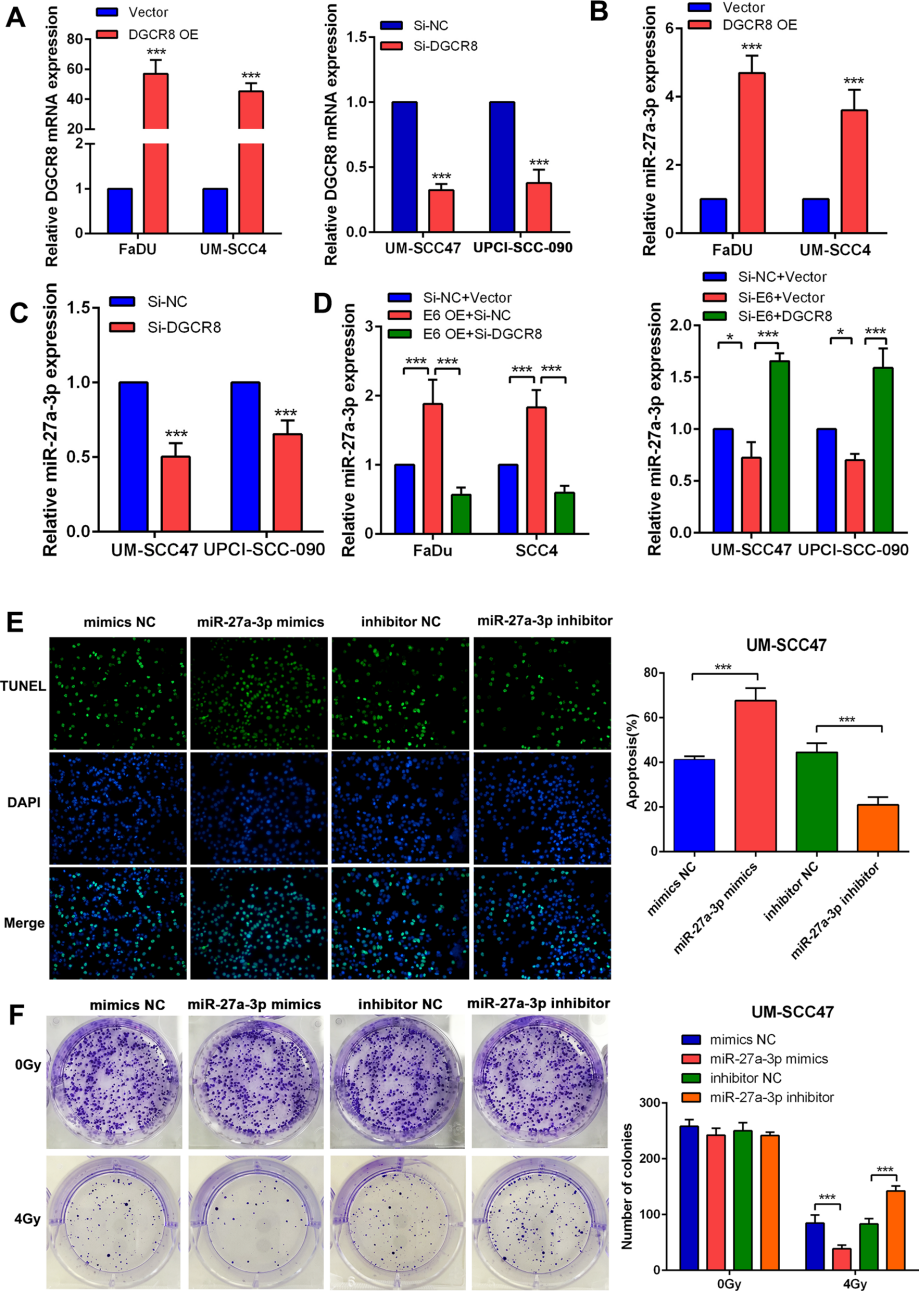
HPV16 E6 upregulated miR-27a-3p via DGCR8 and miR-27a-3p improves radiosensitivity in HNSCC. **a** The relative mRNA expression level of DGCR8 in DGCR8 overexpressing/knockdown HNSCC cell lines. **b**, **c** The relative expression level of miR-27a-3p in DGCR8 overexpressing/knockdown HNSCC cell lines. **d** The relative mRNA expression level of miR-27a-3p in E6-overexpressing HNSCC cell lines with/without DGCR8 knockdown or in DGCR8 overexpressing HNSCC cell lines with/without E6 knockdown. **e** The colonization of TUNEL (+) HNSCC cells under radiation dose of 4 Gy (left panel). The apoptosis rate was evaluated in miR-27a-3p mimics/inhibitor HNSCC cell lines (right panel). **f** Colony formation assay of miR-27a-3p mimics/inhibitor HNSCC cells (left panel). The number of colonies was counted and compared among different groups (right panel). Data were presented as mean ± SD. *P < 0.05; **P < 0.01; ***P < 0.001

To study the regulatory impact of DGCR8 on the expression of miR-27a-3p based on the expression level of DGCR8 in 4 HNSCC cell lines, we generated a DGCR8-overexpressing cell line with FaDu/UM-SCC4 cells and a DGCR8-knockdown cell lines with UM-SCC47/UPCI-SCC90 cells. The overexpression and knockdown efficiencies were evaluated at the mRNA level using RT-qPCR (Fig. 2a). As expected, the expression of miR-27a-3p was upregulated in DGCR8-overexpressing HNSCC cells but downregulated in DGCR8-knockdown HNSCC cells (Fig. 2b, c). Furthermore, knockdown of DGCR8 counteracted the upregulation of miR-27a-3p induced by E6 overexpression, while overexpression of DGCR8 restored the level of miR-27a-3p, which was decreased by E6 knockdown (Fig. 2d). The effect of miR-27a-3p on the radiosensitivity of HNSCC cells was evaluated using a TUNEL assay and a colony formation assay. We observed that after 4 Gy irradiation, the apoptotic activity of HNSCC cells was significantly increased in miR-27a-3p mimics cells compared with control cells, while miR-27a-3p inhibitor resulted in a decrease in apoptosis (Fig. 2e). In the colony formation assay, we found that miR-27a-3p had no effect on the clonogenicity of nonirradiated HNSCC cells. However, under irradiation conditions, miR-27a-3p induced marked death of HNSCC cells (Fig. 2f), suggesting that miR-27a-3p improved that radiosensitivity of HNSCC cells.

### MiR-27a-3p improved the radiosensitivity in HNSCC by targeting SMG1

**Fig. 3:**
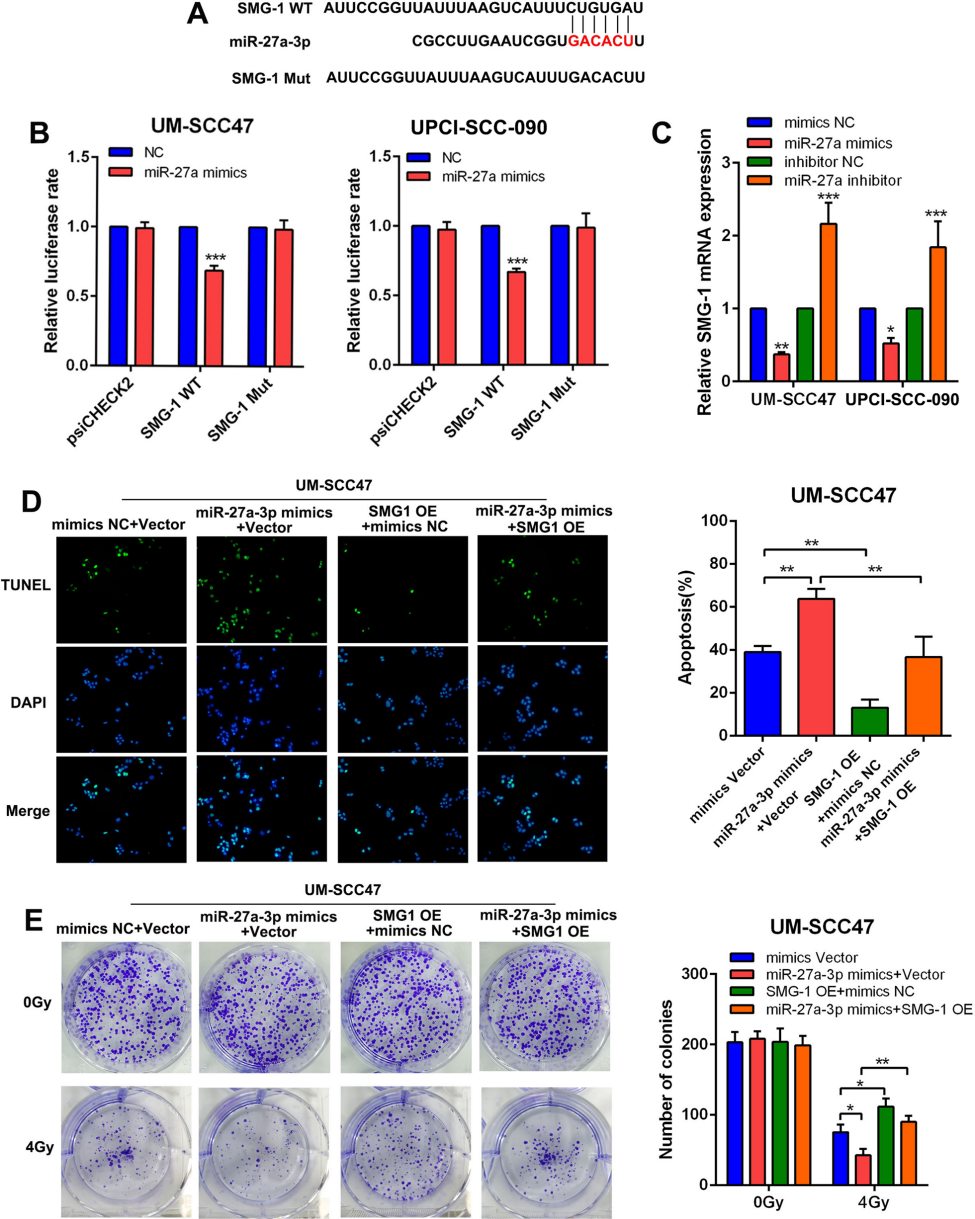
miR-27a-3p improved the radiosensitivity in HNSCC via targeting SMG1. **a** Bioinformatic prediction of the binding site between miR-27a-3p and SMG1. The binding site was marked in red. **b** Dual-luciferase assays were used to detect the luciferase activity in HNSCC cell lines transfected with psiCHECK2, SMG1 WT and SMG1 Mut in the presence of miR-27a-3p mimics. **c** The relative mRNA expression of SMG1 in HNSCC cell lines treated with miR-27a-3p mimics or inhibitor. **d** The colonization of TUNEL (+) HNSCC cells under radiation dose of 4 Gy (left panel). The apoptosis rate was evaluated in SMG1 overexpressing HNSCC cell lines treated with or without miR-27a-3p mimics (right panel). **e** Colony formation assay of SMG1 overexpressing HNSCC cells treated with or without miR-27a-3p mimics (left panel). The number of colonies was counted and compared among different groups (right panel). Data were presented as mean ± SD. *P < 0.05; **P < 0.01; ***P < 0.001

By online bioinformatic prediction tools, we successfully identified that SMG1 directly binds to miR-27a-3p (Fig. 3a). To confirm the binding between miR-27a-3p and SMG1, we constructed recombinant luciferase reporter vectors containing mutated miR-27a-3p binding sites (SMG1Mut). The luciferase reporter assay confirmed the direct binding between miR-27a-3p and SMG1 (Fig. 3b). To study the effects of miR-27a-3p on HNSCC cells, we applied a miR-27a-3p mimic and inhibitor. The expression of SMG1 was significantly suppressed after transfection of the miR-27a-3p mimic but improved after transfection of the miR-27a-3p inhibitor (Fig. 3c). The effect of miR-27a-3p on the radiosensitivity of HNSCC cells was also evaluated using a TUNEL assay and a colony formation assay. We observed that after 4 Gy irradiation, apoptotic activity was significantly increased in HNSCC cells treated with the miR-27a-3p mimic compared with control cells but was decreased in HNSCC cells transfected with the miR-27a-3p inhibitor. However, overexpression of SMG1 counteracted the enhancement of apoptosis induced by miR-27a-3p under irradiation conditions (Fig. 3d). In the colony formation assay, we found that miR-27a-3p had no effect on the clonogenicity of nonirradiated HNSCC cells. However, miR-27a-3p mimic transfection induced marked HNSCC cell death under irradiationunder irradiation, which was counteracted by SMG1 overexpression (Fig. 3e). Collectively, these results indicated that miR-27a-3p improved the radiosensitivity of HNSCC cells by inhibiting SMG1.

### HPV16 E6 affected radiosensitivity in HPV-positive HNSCC by regulating the miR-27a-3p/SMG1 axis

**Fig. 4:**
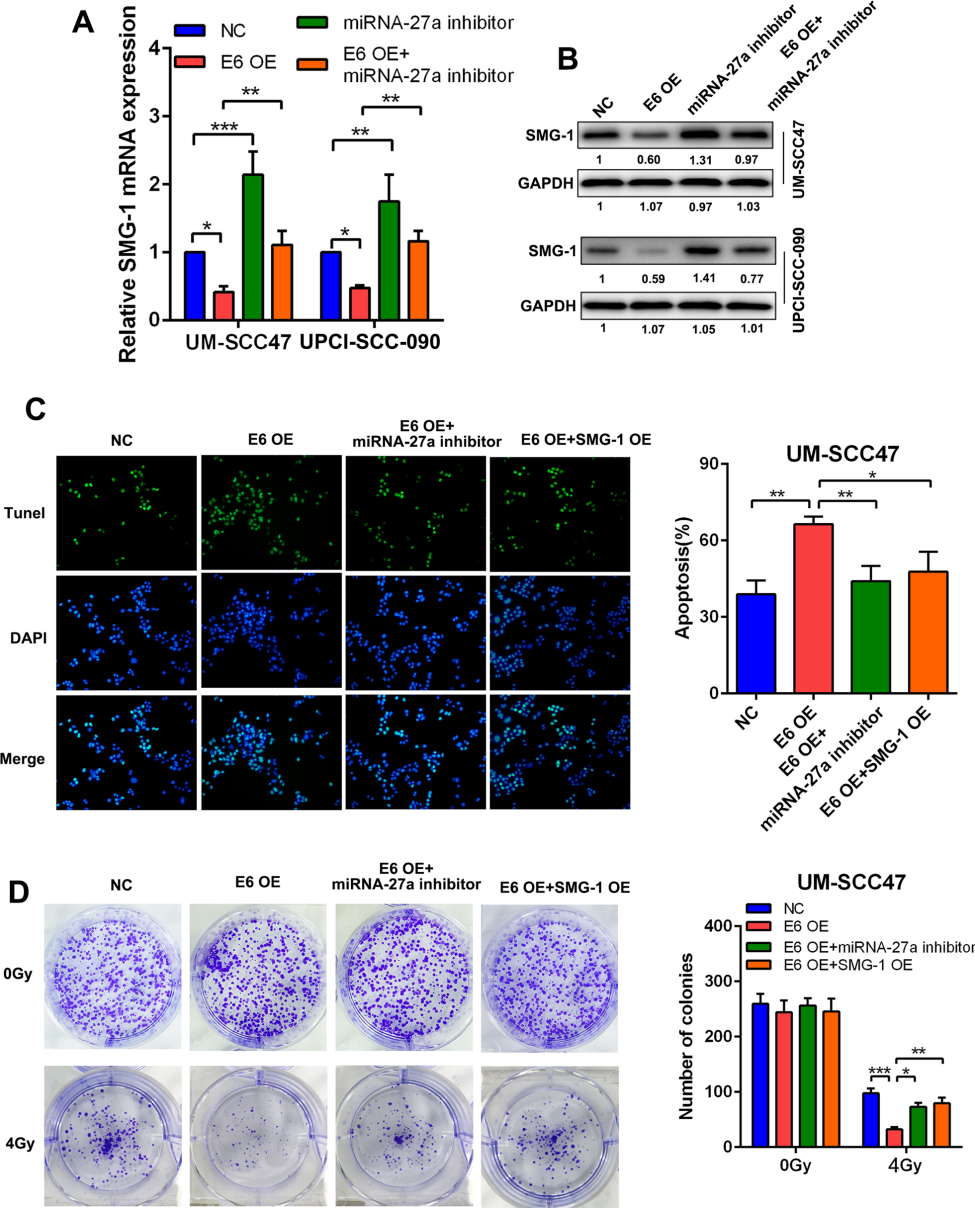
HPV16 E6 affected the radiosensitivity in HPV + HNSCC via regulating miR-27a-3p/SMG1 axis. **a** The relative mRNA expression of SMG1 in E6-overexpressing HNSCC cells treated with or without miR-27a-3p inhibitor. **b** The relative protein expression of SMG1 in E6-overexpressing HNSCC cells treated with or without miR-27a-3p inhibitor. **c** The colonization of TUNEL (+) HNSCC cells under radiation dose of 4 Gy (left panel). The apoptosis rate was evaluated in E6/SMG1 overexpressing HNSCC cell lines treated with or without miR-27a-3p inhibitor (right panel). **d** Colony formation assay of E6/SMG1 overexpressing HNSCC cells treated with or without miR-27a-3p inhibitor (left panel). The number of colonies was counted and compared among different groups (right panel). Data were presented as mean ± SD. *P < 0.05; **P < 0.01; ***P < 0.001

We discovered that the mRNA expression of SMG1 was suppressed in E6-overexpressing HNSCC cell lines, while addition of the miR-27a-3p inhibitor restored the expression level of SMG1 (Fig. 4a). The western blotting results were consistent with the RT-qPCR results (Fig. 4b). In the TUNEL assay, we found that under irradiation conditions, the improved apoptotic activity induced by E6 overexpression was counteracted by miR-27a-3p inhibitor transfection or SMG1 overexpression (Fig. 4c). In the colony formation assay, under irradiation conditions, E6-induced cell death was restored by miR-27a-3p inhibitor transfection administration or SMG1 overexpression (Fig. 4d).

## Discussion

After the discovery of the first oncogenic HPV subtype, HPV 16, viral E6/E7 proteins were identified as key regulators that help sustain the malignant status of HPV-positive cancer cells [28]. Viral E6/E7 proteins are the viral gene-encoded proteins constitutively expressed in HPV-positive cancer cells [29] and the suppression of E6/E7 expression leads to rapid induction of apoptosis, and proliferation arrest in HPV-positive cancer cells [30]. The E6 protein of high-risk HPV strains was known to accelerate the degradation of p53 [31]. Furthermore, the tumor suppressor protein p53 is a major regulator of both G1 cell cycle checkpoints and the death of tumor cells exposed to ionizing radiation and other DNA-damaging agents which lead to DNA strand breaks [32]. In cervical carcinoma, it has been reported that treatment with the corticosteroid dexamethasone increases radioresistance by upregulating viral E6/E7 and that administration of a hormone antagonist (RU486) can reverse this effect [33]. HPV-positive HNSCC carcinoma differs from HPV-negative HNSCC carcinoma not only in tumor biology, but also in clinical features, including its increased radiosensitivity [17, 34]. In recent years, many studies have reported that patients with HPV-positive HNSCC exhibit a better response to ionizing radiation [17]. Expression of wild-type p53, tumor hypoxia and tumor immunology have been revealed as contributing factors to improve radiosensitivity in HNSCC [35, 36].

In a previous study, we found that the mRNA expression of HPV16 E6 was significantly higher in HPV-positive HNSCC than in HPV-negative HNSCC and HPV16 E6 improved the radiosensitivity of HNSCC cells [18].

In the last decade, regulatory functions of microRNAs in cancers have been widely studied [37]. MiR-27a-3p plays different roles in various kinds of malignancies [38, 39]. In colorectal cancer, miR-27a-3p has been reported to be upregulated in tumor tissues and to facilitate tumor progression by inhibiting the tumor suppressor gene BTG1 [38]. MiR-27a-3p has also been found to perform a protumor function in bladder cancer and gastric cancer [40]. Interestingly, miR-27a-3p has been reported to be a tumor suppressor in lung cancer [39], suggesting a complicated connection between miR-27a-3p and cancers. Furthermore, miR-27a-3p has been reported to improve radiosensitivity in esophageal squamous cancer and breast cancer [41, 42]. In this study, we observed that after 4 Gy irradiation, the apoptotic activity was significantly increased in miR-27a-3p mimics HNSCC cells compared with control cells. In addition, under irradiation, miR-27a-3p mimics induced marked death of HNSCC cells in the colony formation assay, which suggested that miR-27a-3p improved the radiosensitivity of HNSCC cells DGCR8 as an oncogene is upregulated in breast cancer, ovarian cancer and glioma [43]. In our study, we found that overexpression of HPV16 E6 could upregulate the expression of DGCR8 in HNSCC while HPV16 E6 knockdown decreased the expression level of DGCR8. DGCR8 is essential for primary miRNAs (pri-miRNA) processing in the cell nucleus and binds to the RNase III enzyme Drosha to form the microprocessor complex that cleaves pri-miRNAs into precursor miRNAs (pre-miRNAs) [44]. Hence, DGCR8 accelerates miRNA maturation. It has been reported that silencing of DGCR8 can decrease the level of miR-27b in ovarian cancer and therefore suppress cancer progression [45]. Via further exploration, we confirmed that both DGCR8 and E6 positively regulated the expression of miR-27a-3p in HNSCC; knockdown of DGCR8 counteracted the upregulation of miR-27a-3p induced by E6 overexpression, while overexpression of DGCR8 restored the level of miR-27a-3, which was decreased by E6 knockdown, suggesting that HPV16 E6 upregulated miR-27a-3p via DGCR8.

Normally, miRNAs perform their regulatory functions by interfering with the expression of their downstream target genes [46]. Via online bioinformatic prediction tools, we successfully determined that SMG1 directly binds to miR-27a-3p which was further confirmed by luciferase reporter assay. In previous studies, downregulation of SMG1 due to promoter hypermethylation was correlated with improved survival in HPV-positive HNSCC.in HPV-positive HNSCC. Via further experiments, we discovered that miR-27a-3p improved the radiosensitivity in HNSCC by targeting SMG1.

In conclusion, our study showed that miR-27a-3p played an important role in HNSCC cells proliferation under the irradiation dose. We demonstrated that HPV16 E6 enhanced SMG1 expression by upregulating DGCR8/miR-27a-3p, improving radiosensitivity in HPV-positive HNSCC. Furthermore, our findings elucidated the clinical significance and regulatory mechanism of HPV16 E6 in HNSCC radiation therapy and provided a prognostic indicator as well as a promising therapeutic target for HNSCC.

## Data Availability

The datasets used and/or analysed during the current study are available from the corresponding author on reasonable request.
